# Identification of Nuclear Protein Targets for Six Leukemogenic Tyrosine Kinases Governed by Post-Translational Regulation

**DOI:** 10.1371/journal.pone.0038928

**Published:** 2012-06-22

**Authors:** Andrew Pierce, Andrew Williamson, Ewa Jaworska, John R. Griffiths, Sam Taylor, Michael Walker, Mark Aspinall O’Dea, Elaine Spooncer, Richard D. Unwin, Toryn Poolman, David Ray, Anthony D. Whetton

**Affiliations:** 1 Stem Cell and Leukaemia Proteomics Laboratory, Manchester Academic Health Science Centre, The University of Manchester, Manchester, United Kingdom; 2 Developmental Biomedicine Research Group, Manchester Academic Health Science Centre, The University of Manchester, Manchester, United Kingdom; University of Leuven, Belgium

## Abstract

Mutated tyrosine kinases are associated with a number of different haematological malignancies including myeloproliferative disorders, lymphoma and acute myeloid leukaemia. The potential commonalities in the action of six of these leukemogenic proteins on nuclear proteins were investigated using systematic proteomic analysis. The effects on over 3600 nuclear proteins and 1500 phosphopeptide sites were relatively quantified in seven isogenic cell lines. The effects of the kinases were diverse although some commonalities were found. Comparison of the nuclear proteomic data with transcriptome data and cytoplasmic proteomic data indicated that the major changes are due to post-translational mechanisms rather than changes in mRNA or protein distribution. Analysis of the promoter regions of genes whose protein levels changed in response to the kinases showed the most common binding site found was that for NFκB whilst other sites such as those for the glucocorticoid receptor were also found. Glucocorticoid receptor levels and phosphorylation were decreased by all 6 PTKs. Whilst Glucocorticoid receptor action can potentiate NFκB action those proteins where genes have NFκB binding sites were in often regulated post-translationally. However all 6 PTKs showed evidence of NFkB pathway modulation via activation via altered IkB and NFKB levels. Validation of a common change was also undertaken with PMS2, a DNA mismatch repair protein. PMS2 nuclear levels were decreased in response to the expression of all 6 kinases, with no concomitant change in mRNA level or cytosolic protein level. Response to thioguanine, that requires the mismatch repair pathway, was modulated by all 6 oncogenic kinases. In summary common targets for 6 oncogenic PTKs have been found that are regulated by post-translational mechanisms. They represent potential new avenues for therapies but also demonstrate the post-translational regulation is a key target of leukaemogenic kinases.

## Introduction

The hematopoietic system can become dysfunctional via various distinct molecular pathologies. For example, the protein tyrosine kinase oncoproteins (PTKs) associated with a number of different leukaemias and preleukaemias have been identified as critical in the molecular pathology of these diseases. They can result from point mutations, reciprocal chromosomal translocations and internal tandem duplication; thus in Anaplastic Large Cell Lymphoma (ALCL) the t(2∶5) translocation leads to the formation of the NPM/ALK gene which encodes a lymphoma specific NPM/ALK protein with intrinsic and disregulated PTK activity [1]. A point mutation in the Kit PTK (D816V) is associated with transformation in mast cell leukaemia [2] and in the myeloproliferative disorders the t(9;22) translocation found within Chronic Myeloid Leukaemia (CML) leads to the production of the BCR/ABL oncogenic PTK [3]. Similarly, Fip1L/PDGFRα PTK is associated with the pathogenesis of about 50% of patients with the hypereosinophilic syndrome [4]. TEL/PDGFRβ PTK is associated with Chronic Myelomonocytic Leukaemia and is the product of a t(9;12) chromosomal translocation [5]. The internal tandem duplication found in Flt3 PTK (Flt3ITD) has been associated with over 20–30% of acute myeloid leukaemia (AML) cases, the Flt3 gaining dis-regulated kinase activity resultant from this insertional mutagenic event [6]. Thus oncogenic PTKs are associated with many hematopoietic malignancies. A key question is therefore, whether these kinases share a common mode of action? Certainly there is some evidence for common activation of canonical pathways such as the Raf/Erk cellular signalling pathway [7].

A key feature of the leukaemias and lymphomas is their progressive nature. The potential reasons for this are numerous. Expression of oncogenic PTKs such as BCR/ABL have been associated with increased genomic instability [8], possibly via increased expression of DNA polymerase β [9]. This may be associated with increased reactive oxygen production [10] that increase the rate of DNA double strand breaks. This attritional rate of DNA modification may then eventually lead to production of clones that have acquired survival/proliferation advantage. Alternatively, post-translational modification of specific proteins may contribute to common pathways for disease progression. The elegant work of Skorski and colleagues has implicated the Nijmegen Break Syndrome protein (Nbs1, involved in DNA repair) as a downstream target for several of the oncogenic PTKs described above [11]. This has been suggested as a mechanism for increased mutation rates. In other words specific changes in the proteome may contribute to disregulation leading to the progressive nature of leukaemia and lymphoma.

Whilst targeted therapies such as the paradigmatic use of imatinib in treatment of CML has altered the landscape in oncology research, the treatment does not eradicate the disease, resistance can develop and the CML stem cell persists [12]. Thus finding novel and common targets in leukaemias and lymphomas may improve treatment strategies.

Methods of finding such aberrations using high throughput, objective screens are now available. In an attempt to identify common downstream targets we have previously described a systematic analysis of a range of leukaemogenic kinases on the whole cell proteome of Ba/F3 cells. Ba/F3 cells were chosen because of their widespread usage in studies on leukemogenesis. Leukaemia generally progresses as cells acquire further mutations within the nucleus due to aberrant signalling cascades. We have shown primitive hematopoietic and embryonic cells alter their proteome with no concomitant change in mRNA [13]. Finding changes in lower abundance nuclear proteins therefore requires subcellular fractionation and proteomic methods. We have analysed the nuclear proteome and phosphoproteome of Ba/F3 cells expressing 6 leukaemogenic PTKs. We demonstrate the importance of post-translational regulation and common targets in glucocorticoid receptor and PMS2 protein expression.

## Results

### Workflow for Relative Analysis of PTK Effects

**Figure 1:**
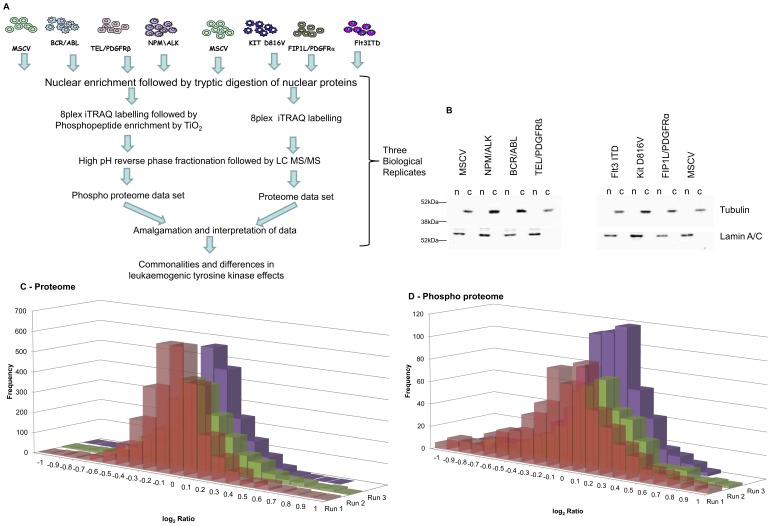
Work flow and quality control. Figure 1a displays a schematic representation of the experimental workflow. Nuclei were enriched from control cells and those expressing the six oncogenes shown on three separate occasions. In each of the three biological replicates the control cells were duplicated to produce an internal technical replicate. The nuclear lysates were then subject to the proteomic workflow illustrated. Figure 1b is a representative western blot of one of the three biological replicate nuclear fractions. Following isolation 7.5 µg of nuclear and 30 µg of cytoplasmic lysates were separated by SDS PAGE and the distribution of lamin and tubulin assessed by western blot analysis. Figure 1c and 1d, Distribution of quantification ratios. These histograms show the distribution of (log) ratios for proteins (Figure 1c - proteome experiments) and phosphopeptides (Figure 1d - phospho peptide experiments).

We have previously described the generation and characterisation of Ba/F3 cells expressing six distinct leukaemia/lymphoma related oncogenic protein tyrosine kinases namely BCR/ABL, TEL/PDGFRβ, Fip1L/PDGFRα, KIT D816V, NPM/ALK or FLT3ITD [14]. Each oncogenically-transfected cell line was shown to be independent of Interleukin 3 (IL-3 is normally required for survival and proliferation of Ba/F3 cells) for growth and have the same growth rate as control (MSCV Ba/F3) cells cultured in IL-3 [14]. In depth analysis of the proteome and phosphoproteome within the nucleus has now been performed using the study design in Figure 1a to assess effectors potentially modulating nuclear function. Subcellular fractionation offered the benefit of multichannel quantification on lower abundance proteins due to decreased analyte complexity.

The quality of the quantitative data required for such a study was ensured by running the experiment outlined in figure 1a three times with biological replicates in extended liquid chromatography/MS analyses. The control cells were present in duplicate in each of the three experiments allowing us to validate the quality of data obtained from 8 channel iTRAQ isobaric tagging in terms of replication and fold change in protein level [14].

Ten million cells yielded 620 µg +/−24 µg of protein (mean+/−sem) with an average nuclear to cytoplasmic ratio of 1∶4 and no significant difference in ratio or protein yield between any of the oncogene-transfected or control cells. In addition to protein yields, lamin and tubulin expression/distribution were used as both loading controls and as a measure of quality of the fractionation (figure 1b).

### Comparative Analysis of the Effect of Expression of Leukaemogenic Tyrosine Kinases on the Nuclear Proteome

The number of proteins detected and relatively quantified with high confidence in the 8 channels was 3619 with a false discovery rate (FDR) of 4.6%. All the data was normalised such that the median was 1.0 and the ratios checked to ensure they had approximately a normal distribution (figure 1c). The total list of proteins identified is shown in Table S1 with all relative quantification values.

**Figure 2:**
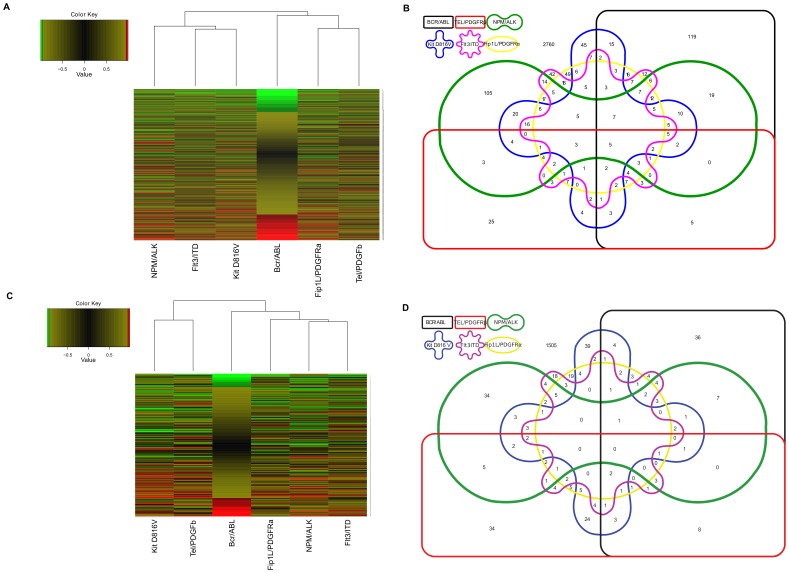
Analysis of potential oncogene relationships. Dendrogram analysis was performed using the standard heat map function of R. Oncogenes are clustered according to similar protein expression profiles observed in iTRAQ ratios. Green indicates a decrease, red an increase and black indicates no change in iTRAQ ratios. The dendrogram tree structure illustrates the relationship between the oncogenes. The varying length of the horizontal “branch” indicates degree of similarity between the oncogenes-a shorter branch indicates a greater degree of similarity. Figure 2a is for the nuclear proteome data and 2c the nuclear phosphoproteome data. An Edwards-Venn diagram was constructed to illustrate the protein changes (figure 2b) and phosphopeptide changes (figure 2d) commonly caused by the oncogenes.

We next defined what constituted a change in protein expression. To be called as changing a protein must have a ratio outside the range in which 95% of protein ratios for the internal replicate are found and a p-value of 0.05 or less in the majority of experiments that a ratio is recorded and not changing in any internal replicate. This “significance interval” was determined for each experimental run and attempts to account for the technical and biological variation seen in each run (see table S1). Protein changes were then examined using a hierarchical clustering (figure 2a). The analysis illustrated that there was very little similarity in the nuclear proteome changes. There was no clustering of the oncogenes involved in the myeloproliferative disorders and whilst FLT3ITD, KIT D816V, Fip1L/PDGFRα (all type 3 leukaemogenic receptor PTKs), did show a greater, though very limited, similarity compared with other oncogenes, TEL/PDGFRβ, another type 3 receptor PTK did not cluster with these structurally similar kinases in respect of proteomic effects. Construction of an Edwards-Venn diagram containing proteins with a change in expression between control cells and each oncogene-transfected cell population also revealed the lack of major overlap in effect between any of the oncogenes (figure 2b). The few commonalities that were identified are shown in table 1, but in general we conclude that leukaemogenic tyrosine kinases have pleiotropic effects on nuclear protein expression. Further informatic analysis did not identify a specific pathway that was significantly affected by all 6 oncogenes via depletion or enhancement of critical but distinct components. Phosphoproteomic analysis also revealed significant differences in modes of action between the oncogenic PTKs using Venn Diagram and hierarchical clustering (Figures 2c and 2d and see section below). It is interesting to note that the hierarchical clustering of the proteome changes and phosphoproteome changes show different patterns. So for example in the proteome changes NPM/ALK, FLT3ITD and KIT D816V cluster together whereas in the phosphoproteome analysis KIT D816V couples with Tel/PDGFβR. Of all the proteins identified, 294 were affected by expression of the NPM/ALK expression, 293, 105, 197 and 214 respectively by the myeloproliferative disorder oncogenes BCR/ABL, TEL/PDGFRβ, Kit D816V, and Fip1L/PDGFRα respectively, and 194 in the FLT3 ITD oncogene found in acute myeloid leukaemia (see table S3 for the list of proteins that change as a consequence of oncogene expression).

**Table 1:** Proteins whose expression is altered by the expression of four or more oncogenes.

Accession	Gene symbol	Protein Name	Change in	BCR/ABL		TEL/PDGFRB		NPM/ALK		Kit D816V		Flt3-ITD		Fip1L/PDGFR	
				Fold change	conf	Fold change	conf	Fold change	conf	Fold change	conf	Fold change	conf	Fold change	conf
ENSMUSP00000046340	Grn	Granulins Precursor (Proepithelin)	6	**1.70**	**0.97**	**1.76**	**0.97**	**1.64**	**0.96**	**1.51**	**0.95**	**1.73**	**0.97**	**1.48**	**0.95**
ENSMUSP00000091374	Zbtb16	Zbtb16 protein	6	**0.32**	**0.99**	**0.53**	**0.98**	**0.55**	**0.98**	**0.66**	**0.95**	**0.50**	**0.98**	**0.55**	**0.97**
ENSMUSP00000117672	Cst3	Cystatin C Fragment	6	**1.57**	**0.99**	**1.37**	**0.96**	**1.71**	**0.99**	**1.49**	**0.98**	**1.38**	**0.97**	**1.41**	**0.98**
ENSMUSP00000028467	Prg2	Bone marrow proteoglycan	6	**0.51**	**0.96**	**0.59**	**0.97**	**0.61**	**0.91**	**0.50**	**0.94**	**0.32**	**0.99**	**0.29**	**0.99**
ENSMUSP00000110538	Hmga1	High mobility group protein HMG-I/HMG-Y	6	**1.85**	**0.98**	**1.63**	**0.96**	**1.87**	**0.97**	**1.89**	**0.98**	**2.82**	**0.99**	**1.97**	**0.98**
ENSMUSP00000027965	Fam107b	Protein FAM107B	5	**0.18**	**0.99**	0.80	(0.71)	**0.52**	**0.99**	**0.53**	**0.98**	**0.58**	**0.98**	**0.70**	**0.88**
ENSMUSP00000050820	Zfp36l2	Butyrate response factor 2 (TIS11D)	5	**1.50**	**0.95**	1.15	(0.67)	**0.58**	**0.97**	**1.64**	**0.96**	**1.66**	**0.97**	**1.51**	**0.95**
ENSMUSP00000016072	Rrbp1	Ribosome binding protein 1	5	**1.78**	**0.98**	1.26	(0.83)	**2.25**	**0.99**	**1.55**	**0.95**	**1.49**	**0.9**	**1.80**	**0.98**
ENSMUSP00000032571	Nova2	Neuro-oncological ventral antigen 2	5	**0.58**	**0.97**	**0.64**	**0.96**	**0.59**	**0.97**	**0.64**	**0.96**	**0.56**	**0.97**	0.74	(0.91)
ENSMUSP00000021471	Tmx1	Thioredoxin-related transmembrane protein 1	5	**1.57**	**0.96**	1.29	(0.83)	**1.64**	**0.97**	**1.52**	**0.95**	**1.71**	**0.97**	**1.59**	**0.96**
ENSMUSP00000055473	Cyb5r3	Putative protein	5	0.80	(0.9)	**1.53**	**0.99**	**0.30**	**0.99**	**0.50**	**0.99**	**0.72**	**0.97**	**1.37**	**0.96**
ENSMUSP00000020365	Mum1	Mutated melanoma-associated antigen 1 (MUM-1)	5	**1.25**	**0.6**	1.11	(0.5)	**2.24**	**0.98**	**1.25**	**0.18**	**1.21**	**0.32**	**0.82**	**0.09**
ENSMUSP00000032182	Xpc	DNA repair - complementing XP-C cells homolog	5	0.75	(0.86)	**0.68**	**0.97**	**0.68**	**0.96**	**0.74**	**0.85**	**0.70**	**0.95**	**0.68**	**0.96**
ENSMUSP00000100653	RP23-383D12	RP23-383D12.6	5	**0.44**	**0.98**	**0.67**	**0.95**	**2.48**	**0.99**	1.06	(0.35)	**1.77**	**0.97**	**0.59**	**0.97**
ENSMUSP00000000769	Serpinf1	Pigment epithelium-derived factor Precursor (PEDF)	5	**0.61**	**0.97**	**0.69**	**0.96**	**0.78**	**0.11**	**0.83**	**0.64**	**0.93**	**0.2**	0.99	(0.14)
ENSMUSP00000094084	Ncf4	Neutrophil cytosol factor 4 (NCF-4)	5	**0.42**	**0.99**	**0.63**	**0.96**	0.91	(0.45)	**0.60**	**0.97**	**0.64**	**0.96**	**0.64**	**0.96**
ENSMUSP00000095099	Sell	Putative protein	5	**0.59**	**0.97**	**1.48**	**0.95**	**1.87**	**0.98**	0.88	(0.63)	**0.65**	**0.95**	**0.56**	**0.97**
ENSMUSP00000118221	Sri	Sorcin isoform 1	5	**1.34**	**0.45**	**1.41**	**0.04**	**2.19**	**0.97**	0.80	(0.8)	**1.75**	**0.98**	**1.38**	**0.03**
ENSMUSP00000021942	Prelid1	PRELI domain-containing protein 1	5	**1.70**	**0.97**	1.42	(0.92)	**2.20**	**0.99**	**2.80**	**0.99**	**2.55**	**0.99**	**1.88**	**0.98**
ENSMUSP00000077335	H2-gs10	MHC class I like protein GS10	5	**1.98**	**0.98**	**1.51**	**0.82**	**1.61**	**0.96**	**2.58**	**0.99**	1.47	(0.94)	**1.56**	**0.95**
ENSMUSP00000060398	Patl1	PAT1 homolog 1	5	**0.48**	**0.98**	**0.67**	**0.95**	0.85	(0.74)	**0.48**	**0.98**	**0.55**	**0.97**	**0.65**	**0.95**
ENSMUSP00000024778	Med20	Mediator of RNA polymerase II transcription subunit 20	5	1.47	(0.94)	**1.56**	**0.96**	**1.58**	**0.96**	**1.66**	**0.97**	**1.75**	**0.97**	**1.48**	**0.95**
ENSMUSP00000026027	Taf5	Transcription initiation factor TFIID subunit 5	5	**0.82**	**0.74**	**0.76**	**0.75**	**0.78**	**0.63**	**0.81**	**0.67**	**0.83**	**0.46**	0.90	(−0.42)
ENSMUSP00000049284	2010321M09	UPF0464 protein C15orf44 homolog	5	**0.89**	**0.19**	**0.77**	**0.58**	**0.84**	**0.4**	**0.85**	**0.34**	**0.80**	**0.27**	0.90	(0.33)
ENSMUSP00000119875	Pms2	Pms2 protein	5	**0.57**	**0.97**	**0.58**	**0.97**	**0.51**	**0.98**	**0.59**	**0.97**	**0.56**	**0.97**	0.72	(0.92)
ENSMUSP00000101533	Nap1l4	Nucleosome assembly protein 1-like 4	5	**1.44**	**0.73**	**1.90**	**0.97**	**1.28**	**0.32**	**1.32**	**0.77**	1.24	(0.51)	**1.31**	**0.69**
ENSMUSP00000023226	Plec	Plectin-1 (Plectin-6)(PLTN)(PCN)	5	**0.45**	**0.99**	0.88	(0.73)	**0.63**	**0.99**	**0.67**	**0.98**	**0.70**	**0.98**	**0.68**	**0.98**
ENSMUSP00000123088	Myef2	Myelin expression factor 2 isoform 1	4	1.10	(0.52)	**1.48**	**0.96**	**1.61**	**0.97**	1.42	(0.93)	**1.51**	**0.96**	**1.59**	**0.97**
ENSMUSP00000028683	Pdia3	Protein disulfide-isomerase A3	4	**1.90**	**0.99**	1.24	(0.7)	**1.57**	**0.9**	**1.50**	**0.95**	1.31	(0.75)	**1.35**	**0.85**
ENSMUSP00000079944	Cox5b	Cytochrome c oxidase subunit 5B	4	**1.40**	**0.36**	1.37	(0.92)	**1.61**	**0.33**	1.32	(0.82)	**1.26**	**0.32**	**1.84**	**0.98**
ENSMUSP00000043559	Cisd1	CDGSH iron sulfur domain-containing protein 1	4	**1.59**	**0.96**	1.36	(0.9)	1.20	(0.63)	**1.51**	**0.95**	**1.59**	**0.96**	**1.99**	**0.99**
ENSMUSP00000075346	AC164613	AC164613.1	4	**1.50**	**0.95**	1.23	(0.82)	**1.64**	**0.97**	1.34	(0.91)	**1.56**	**0.96**	**1.48**	**0.95**
ENSMUSP00000003912	Calr	Calreticulin Precursor	4	1.11	(0.45)	**1.56**	**0.89**	**1.64**	**0.96**	1.27	(0.79)	**1.85**	**0.96**	**1.59**	**0.95**
ENSMUSP00000038329	Nxt1	NTF2-related export protein 1	4	**0.61**	**0.96**	**0.77**	**0.49**	0.89	(0.56)	0.79	(0.83)	**0.72**	**0.84**	**0.78**	**0.69**
ENSMUSP00000071130	Alox5ap	Arachidonate 5-lipoxygenase-activating protein (FLAP)	4	**0.44**	**0.99**	**0.67**	**0.97**	0.98	(0.02)	**0.65**	**0.97**	0.78	(0.86)	**0.74**	**0.92**
ENSMUSP00000002678	TGFβ1	Transforming growth factor beta-1	4	**3.27**	**0.99**	1.10	(0.46)	**1.53**	**0.95**	1.23	(0.78)	**1.65**	**0.97**	**1.67**	**0.97**
ENSMUSP00000033468	Arhgef6	Rac/Cdc42 guanine nucleotide exchange factor 6	4	**0.64**	**0.96**	0.67	(0.94)	0.71	(0.92)	**0.60**	**0.97**	**0.53**	**0.98**	**0.63**	**0.96**
ENSMUSP00000101138	Snx3	Sorting nexin 3	4	**1.84**	**0.98**	**1.49**	**0.95**	1.01	(0.07)	1.20	(0.78)	**1.51**	**0.95**	**1.74**	**0.97**
ENSMUSP00000021077	Slc9a3r1	Na(+)/H(+) exchange regulatory cofactor NHE-RF1	4	**0.41**	**0.99**	0.77	(0.84)	0.85	(0.75)	**0.67**	**0.88**	**0.56**	**0.98**	**0.61**	**0.97**
ENSMUSP00000090256	Heatr7a	HEAT repeat containing 7A	4	**0.63**	**0.96**	1.04	(0.27)	**0.58**	**0.97**	**0.63**	**0.96**	**0.68**	**0.95**	0.88	(0.62)
ENSMUSP00000056774	Pik3r1	Phosphatidylinositol 3-kinase regulatory subunit alpha	4	0.91	(0.58)	0.80	(0.85)	**0.83**	**0.27**	**0.61**	**0.98**	**0.83**	**0.35**	**0.69**	**0.84**
ENSMUSP00000115351	Ttc7	Tetratricopeptide repeat domain 7 Gene	4	0.99	(0.09)	0.98	(0.13)	**1.56**	**0.99**	**1.52**	**0.99**	**1.36**	**0.96**	**0.68**	**0.98**
ENSMUSP00000034881	Cox7a2	Cytochrome c oxidase subunit 7A2,	4	**1.64**	**0.97**	1.44	(0.93)	**1.83**	**0.98**	1.47	(0.94)	**1.54**	**0.95**	**2.00**	**0.99**
ENSMUSP00000015581	Gzmb	Granzyme B(G,H)	4	1.21	(0.73)	**5.65**	**0.99**	**2.12**	**0.99**	**3.41**	**0.99**	1.21	(0.74)	**2.73**	**0.99**
ENSMUSP00000070751	Bsg	Basigin Precursor	4	1.39	(0.93)	**1.52**	**0.95**	1.39	(0.93)	**1.64**	**0.96**	**1.60**	**0.96**	**1.65**	**0.97**
ENSMUSP00000113852	Sykb	Tyrosine-protein kinase SYK	4	**0.05**	**0.99**	0.83	(0.79)	**0.49**	**0.98**	**0.29**	**0.99**	0.73	(0.92)	**0.67**	**0.95**
ENSMUSP00000022904	Atp6v1c1	V-type proton ATPase subunit C 1	4	**0.64**	**0.96**	**0.59**	**0.97**	**0.52**	**0.98**	**0.57**	**0.97**	0.75	(0.9)	0.77	(0.88)
ENSMUSP00000023520	Muc13	Mucin-13 Precursor	4	**1.93**	**0.99**	1.23	(0.88)	**1.74**	**0.99**	**1.35**	**0.95**	0.78	(0.92)	**1.61**	**0.99**
ENSMUSP00000084436	H2–K1	H2-K-alpha-2 gene (haplotype bm9)	4	**1.74**	**0.98**	**1.71**	**0.98**	1.50	(0.92)	**2.14**	**0.99**	1.27	(0.86)	**1.40**	**0.88**
ENSMUSP00000109325	Tpm1	Tpm1 protein	4	**1.89**	**0.98**	1.16	(0.64)	**2.33**	**0.99**	**1.82**	**0.98**	1.31	(0.85)	**1.76**	**0.98**
ENSMUSP00000028848	Fahd2a	Fumarylacetoacetate hydrolase domain-containing protein 2A	4	**1.53**	**0.95**	**1.70**	**0.97**	**1.49**	**0.95**	1.35	(0.91)	1.31	(0.89)	**1.59**	**0.96**
ENSMUSP00000113682	Ccnc	Cyclin C Putative protein	4	0.94	(0.48)	0.82	(0.87)	**1.61**	**0.99**	**1.58**	**0.99**	**1.41**	**0.97**	**0.24**	**0.99**
ENSMUSP00000020238	Hsp90b1	Endoplasmin Precursor (HSP 90 kDa beta member 1)	4	**1.61**	**0.97**	1.21	(0.76)	**1.54**	**0.96**	**1.60**	**0.97**	**1.54**	**0.96**	1.45	(0.95)
ENSMUSP00000023489	Fyttd1	UAP56-interacting factor	4	1.07	(0.06)	1.33	(0.88)	**1.28**	**0.4**	**1.45**	**0.96**	**1.44**	**0.87**	**1.12**	**0.01**
ENSMUSP00000096397	Ehd2	EH domain-containing protein 2	4	**1.68**	**0.99**	1.12	(0.68)	**2.96**	**0.99**	1.27	(0.91)	**3.16**	**0.99**	**1.55**	**0.99**
ENSMUSP00000109190	Fnbp1	Formin-binding protein 1	4	**0.69**	**0.85**	**0.64**	**0.91**	0.85	(0.64)	0.75	(0.87)	**0.71**	**0.85**	**0.71**	**0.88**
ENSMUSP00000065363	Nfil3	Nuclear factor interleukin-3-regulated protein	4	**2.07**	**0.99**	1.07	(0.37)	**1.66**	**0.97**	1.10	(0.46)	**1.82**	**0.97**	**1.92**	**0.98**
ENSMUSP00000088174	Rap1a	Ras-related protein Rap-1A Precursor	4	**1.64**	**0.98**	1.08	(0.31)	**1.94**	**0.96**	**1.37**	**0.94**	**1.24**	**0.35**	1.41	(0.89)
ENSMUSP00000081827	Ptges3	Prostaglandin E synthase 3	4	**1.49**	**0.89**	1.16	(0.22)	**1.27**	**0.61**	**1.41**	**0.87**	1.23	(0.43)	**1.36**	**0.53**
ENSMUSP00000026665	Cbx4	E3 SUMO-protein ligase CBX4	4	**1.56**	**0.99**	1.09	(0.58)	**1.84**	**0.99**	1.01	(0.06)	**2.63**	**0.99**	**1.77**	**0.99**
ENSMUSP00000073371	Lima1	LIM domain and actin-binding protein 1	4	1.27	(0.86)	1.24	(0.83)	**1.54**	**0.95**	**1.71**	**0.97**	**1.53**	**0.95**	**2.19**	**0.98**
ENSMUSP00000022849	Tars	Threonyl-tRNA synthetase	4	**0.51**	**0.98**	0.93	(0.38)	**0.59**	**0.98**	**0.64**	**0.95**	**0.71**	**0.87**	0.74	(0.82)
ENSMUSP00000033995	Rbpms	Putative protein	4	**0.55**	**0.98**	**1.48**	**0.93**	1.23	(0.31)	1.18	(0.44)	**1.50**	**0.96**	**1.56**	**0.96**
ENSMUSP00000110518	Sfmbt2	Scm-like with four MBT domains protein2	4	**0.46**	**0.98**	**0.62**	**0.96**	1.06	(0.33)	**0.58**	**0.97**	**0.59**	**0.97**	0.71	(0.93)
ENSMUSP00000099534	B2m	Beta-2-microglobulin Precursor	4	**2.07**	**0.98**	**2.69**	**0.99**	**2.16**	**0.98**	**3.32**	**0.99**	1.44	(0.94)	1.36	(0.92)
ENSMUSP00000040977	Traf3ip3	TRAF3-interacting JNK-activating modulator	4	**0.64**	**0.91**	0.93	(0.45)	**0.83**	**0.65**	**0.74**	**0.93**	**0.77**	**0.71**	0.76	(0.92)

Proteins shown are those where a confident assessment of a common decrease or increase in 4 or more of the 6 oncogenes has been found. To be included the protein must be called as changing in the majority of experiments in which it is quantified and must not be called as changing in the internal replicate. To be called as changing a protein must have a ratio outside the range in which 95% of protein ratios for the internal replicate are found and a p-value of 0.05 or less. The ratio column contains an average of the ratios across multiple experiments. This is supported by the averaged confidence which indicates how closely the experiments agree. The confidence for each experiment is the proportion of ratios in the internal replicate that the ratio in question is outside of. (So the value for an ‘Up’ call is between 0.95 and 0.99 inclusive). This gives a normalised value between −0.99 and 0.99 for each experiment. A value of greater magnitude indicates more agreement between experiments supporting the change call. Values in brackets in the confidence column are those not called as changing.

### Many of the Common Changes are Neither Due to Protein Translocation nor mRNA Expression Changes

**Figure 3:**
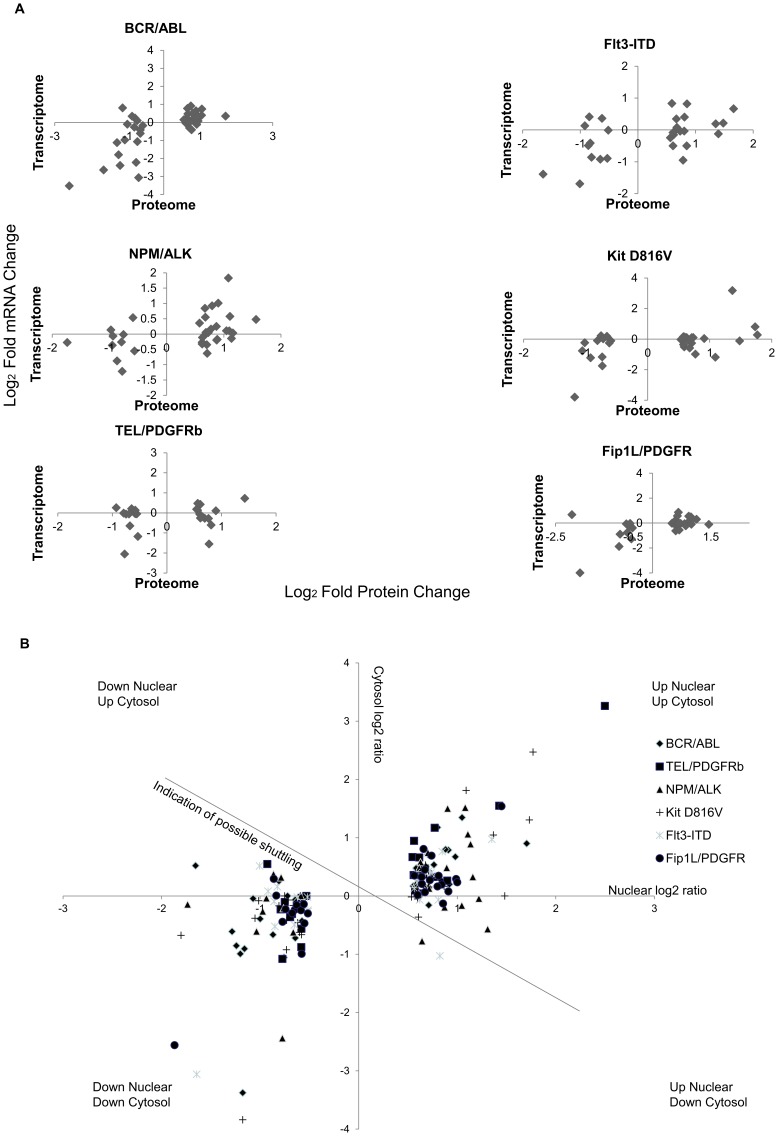
Correlation between protein/mRNA and nuclear/cytosolic protein ratios for proteins identified as changing. Figure 3a; Correlation of mRNA expression changes and protein expression changes for nuclear proteins in response to 6 leukaemogenic PTKs expressed as log2 fold changes. Figure3b; The nuclear and cytosolic changes in protein expression levels (expressed as a ratio value compared to control Ba/F3 cells were plotted for the proteins in which a change of level was seen for four or more of the 6 leukaemogenic PTK transformed cells. Data are shown as Log2 ratios with control mock transfected Ba/F3 cells as denominator.

The lack of overall harmonisation in protein changes emanating from the 6 PTKs led us to look at potential differential layers of control in these systems. To determine whether transcription or post-translational mechanisms were responsible for these changes we compared the data on major protein changes we observed against mRNA expression data we have previously published [14]. This comparison (Figure 3a) demonstrates that there is no direct relationship between change in mRNA expression and proteome change. Having used a nuclear preparation (required to generate data on lower abundance proteins) the reason for this lack of correlation between mRNA and protein change could be translocation of proteins to or from the cytosol. We therefore assessed PTK-induced changes in the cytosol using an equivalent cytosol proteome database (AP, AW, ADW, ST unpublished observations). The change in cytosolic and nuclear levels were plotted (Figure 3b) for the proteins reported to be changing where the proteins were present in both organelles. These data (Figure 3b) clearly show that translocation of proteins was not responsible for the majority of nuclear proteomic changes observed.

**Figure 4:**
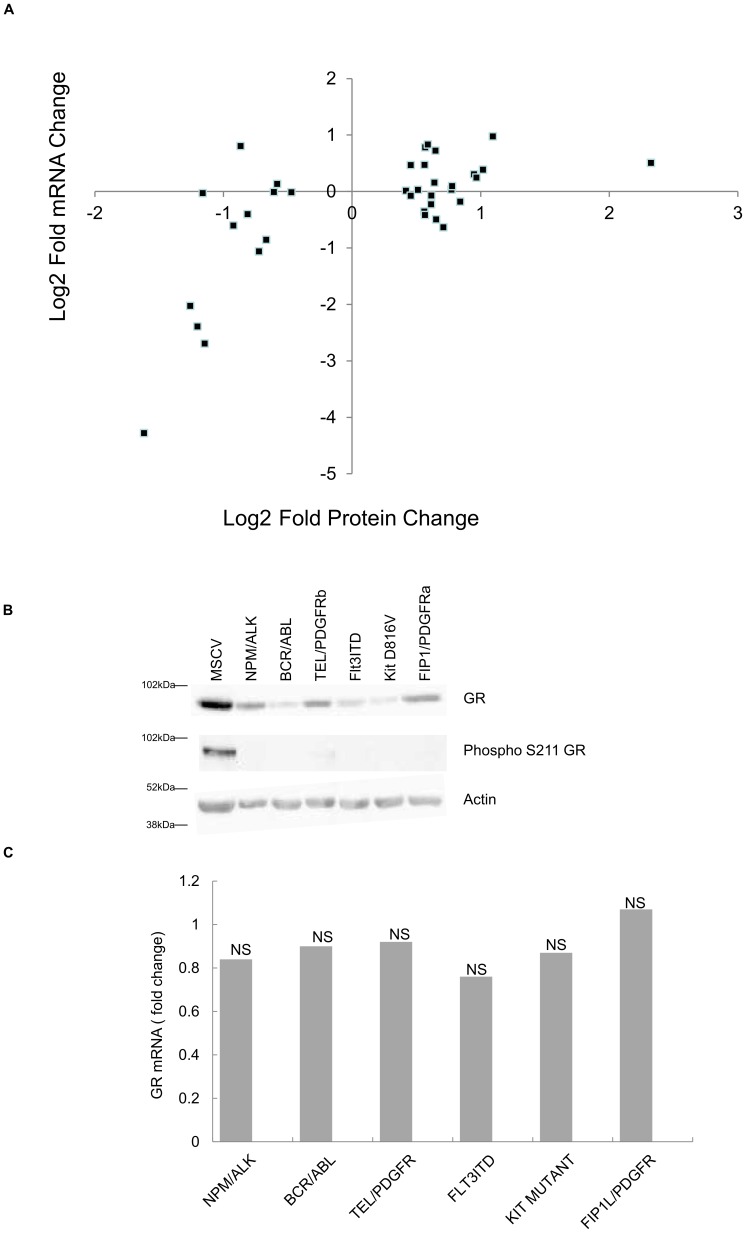
Glucocorticoid receptor changes in response to 6 leukaemogenic PTKs. Figure 4a; The correlation between mRNA level changes and nuclear proteome changes for genes which display a GR binding region were plotted as log2 fold changes with control mock transfected cells as the denominator. Figure 4b; Glucocorticoid Receptor protein levels and the degree of serine 211 phosphorylation of the glucocorticoid receptor in response to expression of the 6 leukaemogenic PTKs were assessed by western blot. Figure 4c illustrates the mRNA expression level changes for the glucocorticoid receptor in response to oncogenic PTK expression, none were statistically significant as measured by students T test.

Given these data we determined if the involvement of any specific transcription factor binding site on genes whose protein levels change gave a strong correlation between protein changes and mRNA changes. In other words do some transcription factors drive an mRNA-mediated change in proteome more readily than others? We analysed the transcription factor binding sites and the most common included NFκB, Fos, Jun, PPAR-γ and Glucocorticoid receptor (GR). One outstanding observation from the consideration of upstream transcription factor binding sites from major changing proteins was the data seen with proteins changing that have upstream glucocorticoid receptors (GRs) binding sites. In no instance did increased protein expression with these gene products correlate with increased mRNA expression (Figure 4a). Where decreased protein expression was observed 38% of genes displayed a >2fold decrease in mRNA levels (comparing control and PTK transfected cells). This led us to examine the levels of GR in PTK and control cells. Figure 4b shows the levels of GR present in PTK expressing cells and their control, mock-transfected counterparts. The GR protein decreased markedly when each leukaemogenic PTK was expressed. Of course, GR was not found in the proteome analysis as it requires activation for nuclear localisation. Further evidence on the lack of activation came from the use of a phospho GR antibody that recognises phosphorylation on Serine 211 (Figure 4b): phospho Ser211 GR levels fell to a greater degree than the levels of GR protein when oncogenic PTKs were expressed. We also recorded that there was no concomitant change in GR mRNA levels (Figure 4c). Thus leukaemogenic PTKs regulate a fundamentally important signal/response coupling pathway post-translationally. Given the use of glucocorticoids to treat leukaemias via induction of apoptosis this is a potentially significant finding. Re-instigation of GR responses may aid in future therapies (see Discussion). Further analysis of upstream pathways modulating GR protein expression is required.

**Figure 5:**
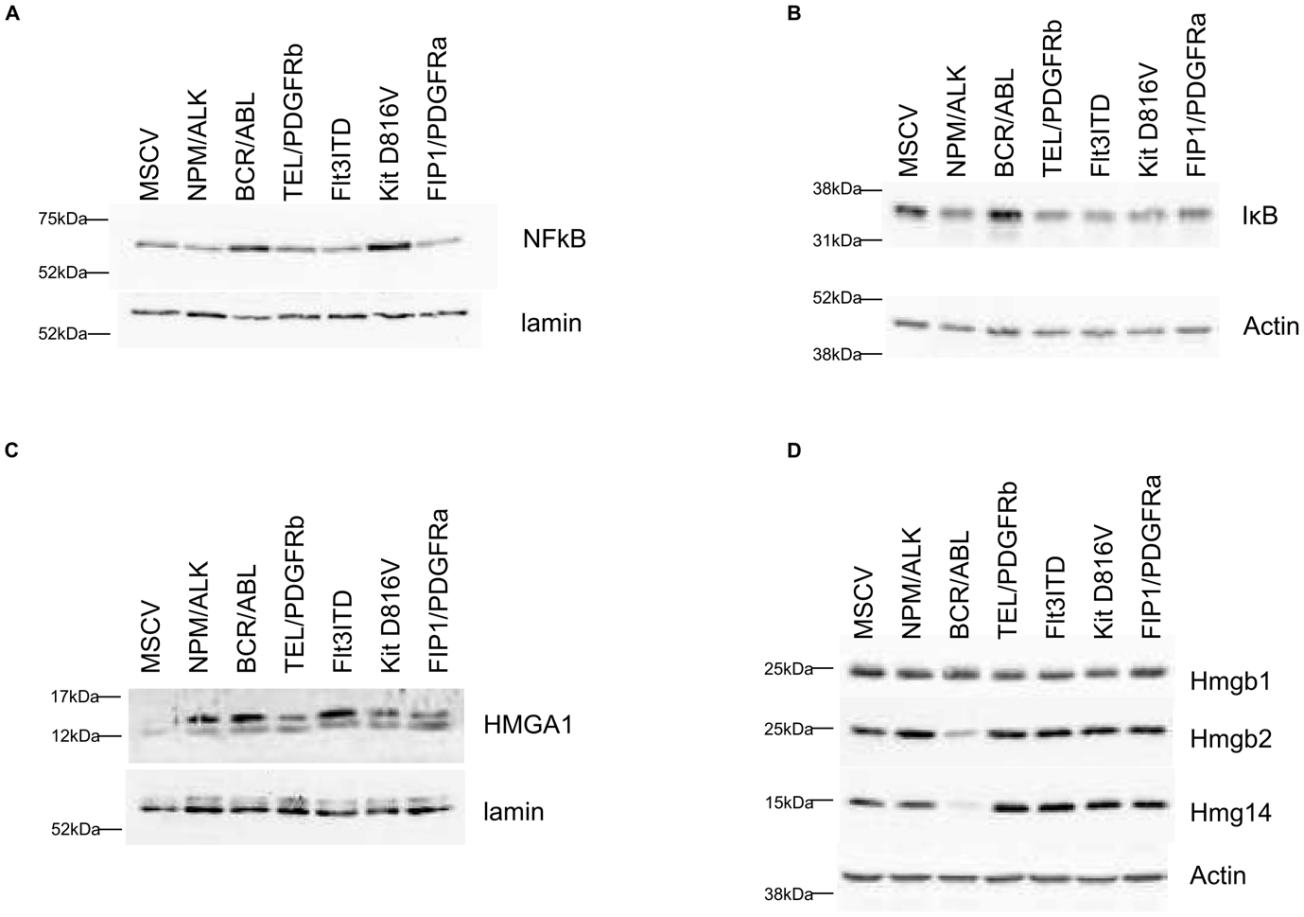
Western blot analysis of NFKb and HMGA1 pathways. Figure 5a to 5d; Cells were lysed or subject to nuclei fractionation before assessment of protein expression by western blot analysis. Expression levels of the proteins shown were assessed in nuclear (figure 5a, 5c) or whole cell lysates (figure 5b, 5d) and protein loading assessed by lamin A/C or actin expression.

NFκB1/2 binding sites were the most common transcription factor binding site found on major changing proteins. This is of interest because NFκB can modulate the GR response and vice versa. IκB levels are a key determinant of NFκB complex activation in cells and all the PTKs with the exception of BCR/ABL decreased IκB (see Figure 5a,b). But a consideration of these protein changes on genes with NFkB binding sites led to the observation that there was no correlative modulation in mRNA where significant protein changes are observed, except in the case of BCR/ABL. With BCR/ABL a two fold modulation of mRNA levels was observed in 50% of proteins whose levels changed in response to expression of this kinase and where NFKB1/2 binding sites are found in the promoter region. This was not the case with any other PTK. As stated all the PTKs studied except BCR/ABL decreased IκB levels, whilst BCR/ABL and KIT D816V increased NFκB protein levels (Figure 5a). We also identified using proteomics the HMGA1 protein which was up-regulated by the oncogenic protein PTKs, this was confirmed by western blot analysis (figure 5c). As a control we showed other HMG family members were not changed as assessed either with mass spectrometry or western blotting (Figure 5d). HMGA1 is an enhancer protein for the NFκB transcription factor complex [15] but with 5 of the 6 oncogenes under analysis this did not affect NFκB regulated gene mRNA synthesis. Taken together these data suggest pleiotropic effects of leukaemogenic PTKS pertain on the NFκB pathway and whilst indicators of activation persist (Figures 5a,b,c and decreased GR expression and activation status) the concept of NFKB addiction in transformation [16] does not apply when transformation is achieved directly and solely from expression of leukaemogenic PTKs. GR level and activity status reduction does not modulate oncogenic PTK-mediated NFκB pathway activation. HMGA1 has multiple effector actions (such as on DNA mismatch repair, see below) which may influence PTK transformed cells with no recourse to NFκB signalling.

### Comparative Analysis of the Effect of Expression of Leukaemogenic Tyrosine Kinases on the Nuclear Phosphoproteome

Approximately 25% of the proteins identified as changing in response to five or six of the PTKs are linked to DNA repair mechanisms e.g. Mum1, Xpc and PMS2. Genomic stability plays a major role in the development and progression of leukaemia [8]. The mechanisms in DNA repair revolve around serine/threonine phosphorylation signalling events. Therefore, in addition to investigating the nuclear proteome we investigated the nuclear phosphoproteome. To achieve this following digestion of nuclear proteins the resultant peptides were enriched on titanium dioxide columns (figure 1a) prior to 2-dimensional LC and mass spectrometry.

We identified a total of 1889 unique phosphoentities, giving approximately 1558 distinct sites of phosphorylation on 705 different proteins (FDR of 0.32%). The amalgamated list is shown in table S4 the total list with precursor mass and charge data in table S5. As with the nuclear proteome analysis biological replicates were employed to determine change in expression with confidence (figure 1c and table S2). The phosphopeptide identified from NF-IL3 illustrated the level of consistency that can be obtained between proteome change and phosphopeptide change. The phosphopeptide ratio compared to control in BCR/ABL, NPM/ALK, FLT3 ITD and Fip1L/PDGFRα were 2.0, 1.8, 2.0 and 1.8 fold respectively. This compares with the data for the protein which gives the following fold changes, 2.1, 1.7 1.8 and 1.9 in other words there was no change in the stoichiometry of phosphorylation on this site.

Phosphopeptides whose expression was altered as a consequence of oncogene expression are shown in table S6. The lymphoma associated oncogene NPM/ALK significantly altered the expression of 91phosphopeptides whilst the myeloproliferative disorder oncogenes BCR/ABL, TEL/PDGFRβ, Kit D816V, and Fip1L/PDGFRα changed the expression of 94, 114, 111 and 68 respectively. In addition 72 phosphopeptides were altered by the FLT3 ITD oncogene associated with acute myeloid leukaemia.

Phosphopeptide changes due to the PTKs were used to classify PTK effects using hierarchical clustering (Figure 2c). As with the nuclear proteome analysis there was very little similarity in PTK-induced changes. Two receptor tyrosine kinases (TEL/PDGFRβ and Kit D816V) did group together though the effect was tenuous. There was no significant pattern to the phosphorylated effectors downstream of the 6 PTKs. This diversity in PTK-induced changes is also illustrated in the Venn diagram shown in figure 2d. A selection of the commonalities that were identified is shown in Table 2. The above data demonstrate there are pleiotropic effects of the PTKs on phosphorylation in the nucleus.

**Table 2:** Phosphopeptides whose expression is altered by the expression of four or more oncogenes.

Phospho Peptide	Protein	Gene Symbol	Name	Change in	BCR/ABL		TEL/PDGFRb		NPM/ALK		KitD816V		Flt3-ITD		Fip1L/PDGFR	
					Fold change	conf	Fold change	conf	Fold change	conf	Fold change	conf	Fold change	conf	Fold change	conf
MDRT[Pho]PPPPT[Pho]LSPAAVTVGR	ENSMUSP00000114916	Phc3	Polyhomeotic-like 3	5	**1.56**	**0.98**	**1.50**	**0.96**	1.23	0.79	**1.69**	**0.98**	**1.45**	**0.95**	**1.49**	**0.96**
LDSSQLPLQTGLDVPAT[Pho]PR	ENSMUSP00000027768	Ahctf1	Protein ELYS (Protein MEL-28)	5	**1.50**	**0.96**	1.20	0.74	**1.44**	**0.95**	**1.60**	**0.98**	**1.47**	**0.96**	**1.57**	**0.98**
DSQDTS[Pho]AEQSDHDDEVASLASASGGFGSK	ENSMUSP00000039134	Edc4	Enhancer of mRNA-decapping protein 4	4	**0.42**	**0.99**	0.95	0.28	**0.58**	**0.98**	1.04	0.18	**0.38**	**0.99**	**0.55**	**0.99**
GPIHS[Pho]PVELQR	ENSMUSP00000065363	Nfil3	Nuclear factor interleukin-3-regulated protein	4	**1.98**	**0.96**	1.27	0.75	**1.75**	**0.94**	1.34	0.75	**2.03**	**0.96**	**1.84**	**0.96**
QSEQPVKPVGPVMDDAAPEDSASPVS[Pho]QQR	ENSMUSP00000106275	Trp53bp1	Tumor suppressor p53-binding protein 1	4	**1.90**	**0.99**	**1.47**	**0.96**	**1.66**	**0.98**	1.41	0.94	**1.48**	**0.96**	1.31	0.87
LQPLTSVDS[Pho]DNDFVTPK	ENSMUSP00000107910	Ncapd2	Condensin complex subunit 1	4	**2.02**	**0.99**	**1.90**	**0.99**	**1.54**	**0.98**	**2.07**	**0.99**	1.17	0.69	0.93	0.36
ELLLDIGDVS[Pho]ER	ENSMUSP00000026448	A2ACJ2	Fanconi anemia-associated protein	4	**0.49**	**0.99**	**0.45**	**0.99**	0.76	0.88	**0.53**	**0.99**	**0.60**	**0.98**	0.75	0.89
S[Pho]PLDNMSR	ENSMUSP00000079818	Etv6	Ets variant gene 6 (TEL oncogene)	4	1.34	0.89	**1.52**	**0.97**	**1.77**	**0.99**	**1.80**	**0.99**	**1.56**	**0.98**	1.34	0.89

Phosphopeptides shown are a selection where a confident assessment of a common decrease or increase in 4 or more of the 6 oncogenes has been found. To be included the peptide must be called as changing in the majority of experiments in which it is quantified and must not be called as changing in the internal replicate. To be called as changing a peptide must have a ratio outside the range of which 95% of phosphopeptide ratios for the internal replicate are found. The ratio column contains an average of the ratios across multiple experiments. This is supported by the averaged confidence which indicates how closely the experiments agree. The confidence for each experiment is the proportion of ratios in the internal replicate that the ratio in question is outside of. (So the value for an ‘Up’ call is between 0.95 and 0.99 inclusive). This gives a normalised value between −0.99 and 0.99 for each experiment. A value of greater magnitude indicates more agreement between experiments supporting the change call. Values not in bold are those not called as changing (details can be found in the supporting information tables).

### Identification of Common Downstream Nuclear Targets for the Leukemogenic PTKs

Proteins previously associated with the development of leukaemias, such as hnRNP1 [17] and SHIP1 [18] the inositol lipid phosphatase, show altered phosphorylation as a consequence of the downstream action of several PTKs (table S6). A selection of the phosphorylation events most commonly seen between the 6 PTK expressing cells are shown in Table 2. P53bp1 interacts with the tumour suppressor protein p53 and has a role in DNA damage response [19]. Its phosphorylation is affected by 4 of the PTKs in the study but the oncogenes do not affect the expression level of this protein. It has previously been reported that p53bp1 S1099 is a phosphorylated downstream from Flt3 ITD [20]. We have now demonstrated that in addition it is phosphorylated by several of the PTKs studied here (namely BCR/ABL, NPM/ALK and TEL/PDGFRβ and Flt3 ITD). Decreased levels of Serine 211 phosphorylation on GR (Figure 4b) in response to leukaemogenic PTK expression are far in excess of the decrease in protein levels (in other words there is a decreased stoichiometry of phosphorylation as well as a decreased GR protein level). This phosphorylation is likely governed by serum supplement derived GR agonists. The configuration of proteomic/mRNA changes observed in our study led to this observation that can now be further analysed for potential clinical relevance.

Similarities in changes in the protein level are shown in Table 1. Cystatin C and bone marrow proteoglycan both change in response to expression of all 6 PTKs. We have generated a nuclear preparation free from cytosolic protein contamination and extracellular medium which is illustrated by the lack of serum proteins and key cytosolic proteins (see complete list in table S1 and figure 1b). Therefore one can infer these proteins are indeed associated with nuclei. Given the role of cystatin C as a lysosomal protein inhibitor there may be disregulation of lysosomal function in PTK transformed cells potentially associated with disregulated autophagy [21]. This is associated with inflammatory conditions, a theme we extend below. Zinc Finger and BTB binding domain protein 16 (ZBTB16) is a cell cycle regulatory transcription factor [22] decreased in expression by 6 out of 6 PTKs in our study, this protein associates with key regulators of hematopoiesis such as GATA2, BMI1 and PML [23], [24]. The protein showing the greatest level of up-regulation was High Mobility Group Protein A1 (or Y). Perhaps surprisingly TGFβ is upregulated in four of the six oncogene transfected cell lines. Elevation by leukaemogenic PTKs is also seen in the cytosol (AP, ST, ADW, unpublished observations). The importance of TGFβ in the natural history of CML is highlighted in a recent paper by Naka et al. [25] which reported that inhibition of TGFβ impaired colony formation in CML and proposed a role for TGFβ inhibition in CML treatment. Given these important observations oncogenic PTK-transfected and control cells were treated with the TGFβ receptor kinase inhibitor LY364947 and viability assayed over 3 days. No differences in response were seen between PTK transfected and control cell lines (figure S1).

NF-IL3 (E4bp4) is up-regulated by the action of 4 out of 6 PTKs (Table 1) a target of IL-3 it plays an important role in survival; indeed over-expression of NF-IL3 leads to IL-3 independence in Ba/F3 cells [26]. This suggests that the oncogene-induced increase in NFIL-3 expression may play a role in the oncogene expression related growth factor independence seen in the Ba/F3 cells with respect to BCR/ABL, NPM/ALK, FLT3 ITD and Fip1L/PDGFRα.

### PMS2 a DNA Mismatch Repair Protein is Down Regulated by Leukaemogenic Protein Tyrosine Kinases

**Figure 6:**
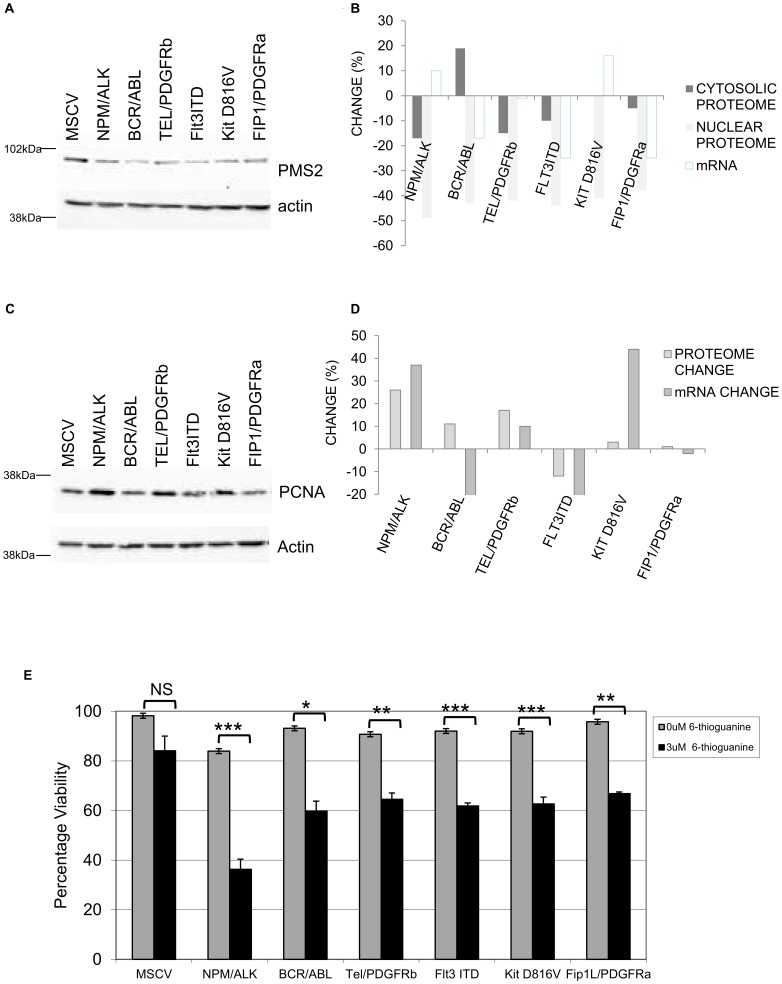
Regulation of the expression of DNA mismatch repair protein PMS2. Figure 6a; PMS2 expression levels were assessed by western blot analysis in whole cell lysates and actin expression used as a loading control. Figure 6b shows the Isobaric tagging tandem MS relative quantification data for PMS2 levels in the nucleus and cytosol and comparison of these values to mRNA changes observed for PMS2. Figure 6c; PCNA expression levels were assessed by western blot analysis in whole cell lysates. Actin expression was used as a loading control. Figure 6d; isobaric tagging tandem MS relative quantification of nuclear PCNA levels and comparison of these values to mRNA values for PMS2. Figure 6e; Control and leukaemogenic PTKs transfected Ba/F3 cells were cultured with mIL-3 in the presence or absence of 6-thioguanine (3 µM) for 24 hours and viability assessed by trypan exclusion. The results of a t-test between treated and untreated are shown and represented by; * <0.01, ** <0.005, *** <0.001.

Another protein fundamentally linked to DNA repair and markedly decreased (by >40% in the case of all PTKs except Fip1L/PDGFRα (which gave a 28% though non-significant reduction) was PMS2. We validated this observation by western blot analysis (figure 6a). This demonstrated that all 6 leukaemogenic PTKs decreased PMS2 levels. Whether this was due to translocation to the cytosol was also assessed. An isobaric tag-based MS analysis of the cytosol proteome of the 6 leukaemogenic PTK-transformed cells showed the presence of PMS2 but no alteration in its levels (Figure 6b). Similarly mRNA levels for PMS2 were not altered in a significant fashion. The observed decrease in the nuclear PMS2 level is due then to mechanisms other than transcriptional or protein shuttling mechanisms. To confirm the specificity of this PMS2 effect another member of this DNA mismatch repair complex (PCNA) was assessed by western blot and shown to be unaffected or increased. A potential increase of the protein level mediated by NPM/ALK and TEL/PDGFβR was seen using iTRAQ (though this was below our threshold cut off level) and by western blot, there was no correlation of any change with mRNA changes (figure 6c,d).

DNA mismatch repair plays an important role in maintaining genomic stability. Given our proteomic findings that pathways that potentiate genomic stability are common targets mediated by 6 oncogenic PTKs we investigated whether such pathways were perturbed in the oncogene expressing cells. Cells were treated with the DNA mismatch repair inducing drug 6-thioguanine and viability assessed 24 hours later. As is clearly illustrated in figure 6e the oncogene expressing cells have a defect in their DNA repair pathway showing a reduced viability following treatment. This indicates that the oncogene expressing cells are less able than the control cells to repair the DNA mismatches induced by the 6-thioguanine.

**Figure 7:**
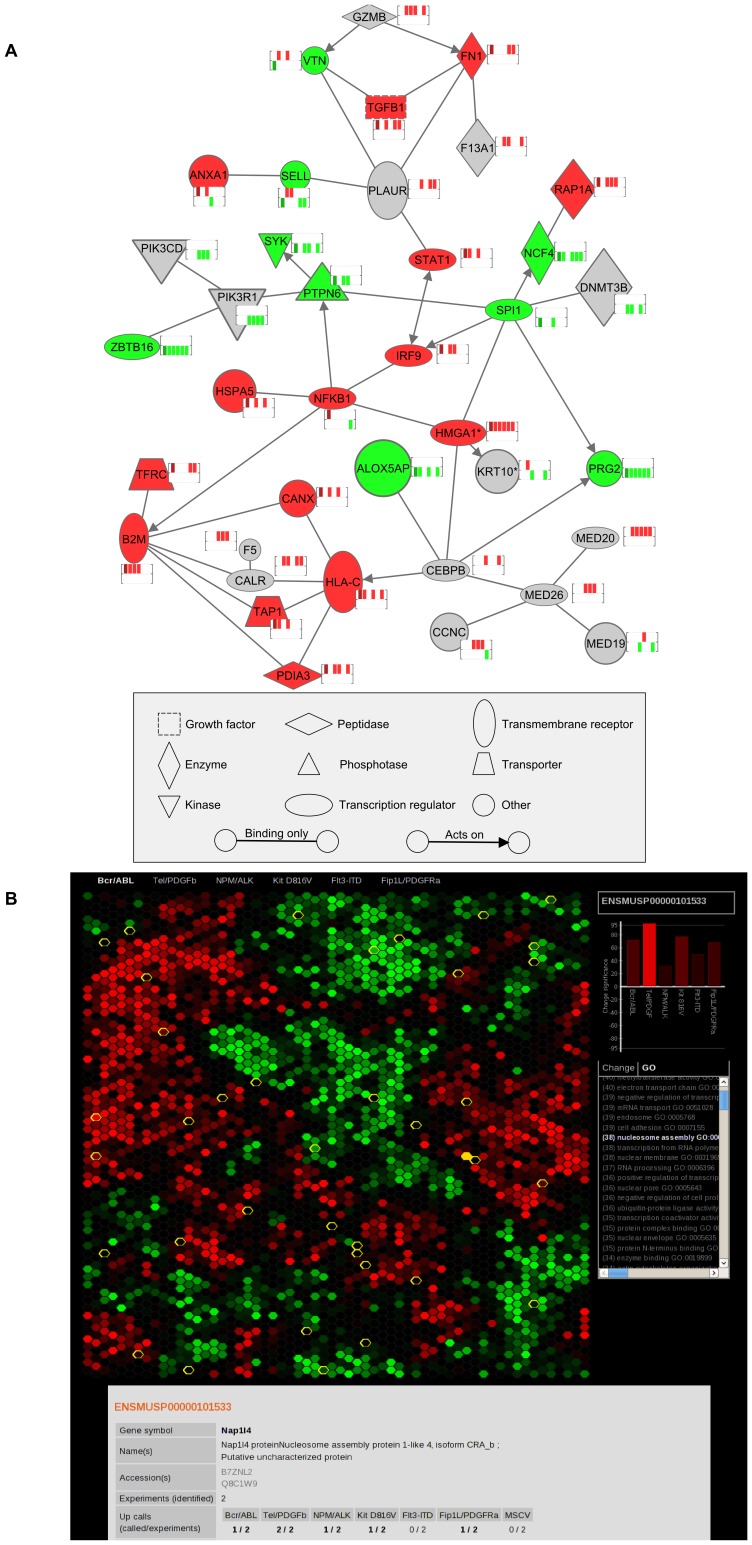
Schematic analyses of proteomic data. Figure 7a: Ingenuity pathway analysis software was used to examine the HMGA1 interaction network within the differentially regulated proteins/phospho-peptides regulated by oncogenic PTKs found in the study. Immediate interactors that are differentially regulated in at least two oncogenes are shown while further interaction relationships are shown where differential regulation is indicated in three or more oncogenes. The mini-heatmaps show the regulation seen in the six oncogenes. Red indicates up-regulation and green down-regulation. From left to right the six columns show BCR/ABL, TEL/PDGFRβ, NPM/ALK, Kit D816V, Flt3-ITD and Fip1L/PDGFRa. Figure 7b; A screen snap of the interactive web-based viewer used to display the data. The web site can be found at http://www.scalpl.org/scope3/.

The data on major protein changes and their interactions is shown in Figure 7a. Furthermore, all protein changes have been developed into a web-based system for checking proteins of interest, depicted in Figure 7b.

## Discussion

Our substantial analysis of the effects of 6 leukemogenic PTKs has revealed a great deal of disparity in their effects in the nucleus, a novel and important observation in itself. We then went onto show that modulation of mRNA levels and nuclear/cytosolic shuttling were not responsible for the major changes seen in the nuclear proteome instigated by the leukaemogenic PTKs. The very fact that translocation and mRNA modulation do not account for many of the changes observed underlines the need for organellar proteomic analyses to identify the targets downstream of oncogenic PTKs and also the need for further research on mRNA processing and translation regulation plus protein hydrolysis mediated by leukaemogenic PTKs.

A key example of a changing protein is HMGA1. This protein has been shown to be relevant in the leukaemias showing increased expression in a major proportion of Acute Myeloid Leukaemia cases [27]. This non-histone protein is highly expressed during embryogenesis and involved in many cellular processes, including regulation of gene transcription and potentially DNA damage via its interaction with BRCA1 [28]. Its role in transformation has been documented extensively elsewhere: HMGA1 is elevated in poorly differentiated carcinomas where its expression is related to a poor prognosis; HMGA1 induces oncogenic transformation in cultured cells and causes lymphomas in transgenic mice (for a review see [29]). Also cells over-expressing HMGA1 exhibit increased UV sensitivity due to effects on inhibition of nucleotide excision repair. XPC protein, a component of the nucleotide excision repair pathway involved in correction of G/T mismatches [30]. Western blot analysis confirms the iTRAQ data (figure 4d) that HMGA1 specifically amongst HMG family members is up-regulated by all the six oncogenes. In our experiments we have shown HMGA1 elevation is not associated with increased NFκB responsive gene expression despite its role as an enhancer for the NFκB complex.

Other interactors of HMGA1may therefore also be important. HMGA1 can modulate the response to insulin and to interact with the key hematopoietic regulator C/EBPβ [31]. Elevated expression of cytokines like IL-3 and Kit ligand are a part of the pathogenesis of diseases such as Chronic Myeloid Leukaemia, the proven ability of increased HMGA1 expression to bind the Kit ligand promoter and enhance expression thereof adds another facet to the pleiotropic effects of elevated HMGA1 expression [32].

As described above, GR gene targets are potentiated by the 6 PTKs and the levels of this key receptor are decreased markedly by 6 out of 6 of the leukaemogenic PTKs. Phosphorylation on a GR site associated with activation [33] is also decreased. The GR agonists have been implicated in regulation of erythropoiesis [34] and are required for optimal maintenance of hematopoietic primitive cells in long term marrow cultures [35]. Glucocorticoids are employed to great effect in the treatment of some leukaemias [36], [37]. Thus the observation that leukaemogenic PTKs decrease expression levels of GR has potential clinical implications. Acquired steroid resistance is a serious clinical issue [38], [39], [40] that can affect outcome. Acquired mutations may be responsible for this and now we have some indication that signal transduction pathway activation can underlie this. In our model system we can investigate upstream events that account for the observed GR down-modulation. In so doing we can identify potential therapeutic strategies to instigate a response to steroid treatment that could lead to leukaemic cell apoptosis.

A study on the occurrence of mutations in two proteins involved in DNA mismatch repair, MSH2 and MLH1, has previously implicated alterations in this pathway in AML pathogenesis [41]. Here we demonstrate that PMS2, another protein involved in DNA mismatch repair is altered by oncogene expression. PMS2 has previously been reported to be associated with Lynch Syndrome [42] and colorectal cancer [43]. PMS2 recessive mutations have been associated with leukaemias [44]. It is a downstream target of p53 and is believed to sense DNA damage enabling decisions on repair versus commitment to apoptosis [45]. We reasoned that the reduced levels observed in the wake of expressing oncogenic PTKs would likely alter the balance between survival and apoptosis in PTK expressing cells. This indeed was the case as oncogene-transfected cells subjected to 6-thioguanine treatment, a drug which induces DNA mismatches, were less able to survive compared to their normal counterparts. Decreased response to drug is seen where PMS2 deletion occurs and in this context the results we observe could be said to be counterintuitive. However other data from PMS2 heterozygotic animals show this is not the case. It is clear that PMS2 null mice, and specifically rates of mutation therein, are dramatically different with PMS2+/− mice [46]. In the effects of the 6 oncogenic PTKs we see approximately 50% reduction in PMS2. This will not necessarily generate the lack of response seen in PMS2 null human disease and mouse models [44], [47]. Rather the subtle but still statistically significant effect we observe in response to thioguanine in the presence of the leukaemogenic PTKs is more in keeping with the literature on PMS2 heterozygosity [46]. Thus our endeavour to identify mechanisms of control for the nuclear proteome and triangulate a common nuclear effect of 6 oncogenic PTKs from lymphoma, AML and MPDs has yielded a target that may be of benefit in the clinic. This PMS2 observation could not have been made with mRNA measurements demonstrating the need for proteomics-based discovery. Furthermore, there is a differential response to thioguanine and as such the use of drugs such as PolyADP Ribose Polymerase in combination with chemotherapeutic agents that invoke DNA mismatch repair may increase leukaemic cell kill.

Protein phosphorylation effectors of the oncogenic PTKs is likely in part explained by their differential subcellular localization as well as pathways activated in the cell. Choudhary et al [20] show that Flt3-ITD expressed in different parts of the cell affects different signalling pathway activation. They also demonstrate that there is a common effector pathway between the different Flt3ITD species examined. This group also note the phosphorylation of Polyhomeotic homolog 3 by Flt3ITD, this is a target found in our studies (affected by 5/6 PTKs) that modifies chromatin via monoubiquitination of histone H2A. Clearly the oncogenic PTKs studied promote further rounds of post-translational modifications. ELYS is also a major downstream target of the oncogenic PTKs (Table 2), a protein involved in chromosome/nuclear pore complex machinery that has a role in mitosis, post-translational regulation may promote inappropriate events during cell division. Fanconi Anemia associated Protein phosphorylation is down modulated by leukaemogenic PTKs, this protein can regulate Fanconi protein repair complex specifically via FANCD2 ubiquitination [48]. P53 Binding Protein 1 shows increased phosphorylation in the PTK-transfected cells, this post-translational modification is also associated with DNA damage [49]. Thus there is potentiation of DNA damage repair pathways on a number of levels.

Our results are summarised in figure 7a in respect of pathway analysis of effects observed. We conclude that our study, by taking cell cycle status out of the equation, assessing protein translocation to the cytosol and mRNA changes for key nuclear protein changes has highlighted post-translational control as key in leukaemogenic PTK action and identified several targets that are common for these PTKs. This idea of post translational control was recently highlighted in a study of protein tyrosine phosphatases (PTP) activity in normal and oncogenic signalling [50]. Kariash et al were able to demonstrate that cancer cells display distinctive patterns of PTP activity revealing additional levels of complexity in the regulation of protein-tyrosine phosphorylation in normal and malignant cells. In addition they were able to demonstrate regulation of phosphatase activity via ROS which could link this phenomenon through to leukaemic oncogenic PTKs which are known to increase ROS [51]. These data offer reasons for the complexity seen in the effects of the 6 oncogenic kinases we have studies. However the approach we have employed offers only a picture of the equilibrium phosphopeptide status and further work will be required to identify if this is modulated by protein kinase activity change or protein phosphatase activity change. The repository provided from our screen will be of value to those studying a number of distinct leukaemias and is accessible through user friendly software (Figure 7b) http://www.scalpl.org/scope3/. Further validation studies will identify potential targets for therapy in PTK-mediated malignant disease.

## Materials and Methods

### Cell Lines

Ba/F3 cells [52] were transfected with either an “empty” MSCV retroviral vector, or MSCV containing BCR/ABL, TEL/PDGFRβ, Fip1L/PDGFRα, KIT D816V, NPM/ALK or FLT3ITD and maintained as stable cell lines as previously described. [14] The resultant cell lines were maintained in culture in Fischer’s medium (Invitrogen, Paisley, UK) with 10% (v/v) horse serum (Biowest, Nuaillé, France). Ba/F3-MSCV cells were grown in Fischer’s medium with 10% (v/v) horse serum supplemented with 5% (v/v) mIL-3 (conditioned media from X63-Ag-653 cells).

### Preparation of Cellular Nuclei and Western Blot Analysis

Nuclear proteins were enriched using a kit from Active Motif (Rixensart, Belgium) with some modifications. Briefly, 1×10^7^ cells were washed in ice cold Hanks buffer with protein phosphatase inhibitors and incubated in hypotonic buffer (750 µl) as per manufacturer’s instructions. The preparation was then incubated on ice for 20 minutes in 50 µl TEAB (0.5 M), 0.05% (w/v) SDS and 0.5 µl of protease inhibitor cocktail (Active Motif) and 2 µl/ml Benzonase (Novagen, UK). The preparations were mixed, centrifuged for 10minutes at 14,000 g and the supernatant (nuclear fraction) recovered. The quality of the preparations was confirmed using western blot analysis for nuclear (lamin A/C) and cytosolic (α tubulin) standard marker proteins.

Whole cell lysates were prepared by lysing cells in RIPA buffer (50 mM Tris pH 7.4, 1% NP-40, 0.25% Na-deoxycholate, 150 mM NaCl and 1 mM EDTA). Briefly cells were washed three times with Phosphate buffered saline before lysing in RIPA buffer with the following additions; Protease Inhibitor Cocktail (Sigma-Aldrich, cat. #P8340), Phosphatase Inhibitor Cocktail 2 and 3 (Sigma-Aldrich, cat #P5726 and #P0044) and sodium orthovanadate (1 mM). Western blot analysis was carried out by standard protocols. Antibodies used were actin (A5060), HMGA1 (SAB2501305) from Sigma (Poole, UK); α tubulin (sc-5286), NFκB (sc-372), IκB (sc-371) and Glucocorticoid receptor (sc-1004) from Santa Cruz (Wembley, UK); Lamin A/C (# 2032) Phospho Ser211 Glucocorticoid Receptor (#4161) from Cell Signalling Technology (Hitchin, UK); HMGb1 (ab18256), HMGb2 (ab11973), HMG14 (ab5212) from Abcam (Cambridge, UK); GSK3 phospho Y216 (612312), PCNA (610664) and PMS2 (556415) from Becton Dickenson (Oxford, UK); HSP90b (RB-118-PO) from Thermo Scientific (Runcorn, UK); GSKα/β (KAM-SToo2) from Enzo Life Sciences (Exeter UK).

### Work Flow

The work flow is outlined in figure 1. Nuclei were enriched from control cells (MSCV) and those expressing the six oncogenes on three separate occasions to provide three biological replicates. In each of these three biological replicates the control cells were duplicated to produce an internal replicate.100 µg of nuclear proteins from each cell line were reduced, alkylated and subject to tryptic digestion prior to labelling with 8 channel isobaric tagging ITRAQ™ reagent in 1 M TEAB, according to the manufacturer’s instructions (in all experiments isobaric labelling exceeded 98% of total identified peptides using mass spectrometry, see below). Isobarically tagged peptides were then taken forward for analysis or subject to phosphopeptide enrichment on TiO_2_ columns. The ensuing nanoflow liquid chromatography plus tandem mass spectrometry were performed as described previously [14] and are in further detailbelow. Mass spectrometry data were processed using Protein Pilot software 3 (ABSciex, USA). The experiment (including generation of cell pellets) was performed three times for both proteome and phosphopeptide assessment. A change in protein level is defined as a ratio where the p-value is not more than 0.05 and the ratio is outside of 95% of all ratios for the internal replicate. A change in phosphopeptide level is defined as a ratio that is outside of 95% of all ratios for the internal replicate. The ratios for these confidence ‘intervals’ for all the experiments are shown in table S2.

### TiO_2_ Mediated Enrichment of Phosphopeptides

100 µg of nuclear iTRAQ labelled peptide mixtures were re-suspended in 150 µl of lactate loading buffer (240 mg/mL lactate in 80% (v/v) acetonitrile, 1% (v/v) Trifluoro-acetic acid (TFA). TiO_2_ columns (TopTip, Glygen Corp, USA) were equilibrated with 150 µl of lactate loading buffer then the samples loaded. The tip was washed twice with 60 µl of lactate loading buffer and four times with 60 µl of wash buffer (80% (v/v) acetonitrile, 5% (v/v) TFA). Bound peptides were then recovered by elution in 60 µl of elution buffer (ammonium water [20 µl NH_3_ in 980 ul H_2_O], pH 10.5). Samples were then concentrated to a few µl in a SpeedVac prior to RP-LC-MS/MS as above.

### High pH Reversed Phase Chromatography

Prior to reversed phase LC-MS/MS peptides were fractionated off line using a reversed phase chromatography column (Fortis technologies, C18 3 µm 100/4.6 Reversed phase column) at a high pH using an LC Packings Ultimate LC system. The gradient was run at 700 µl/min using initially 99.5% high pH buffer A (0.1% Ammonium hydroxide, adjusted to pH 10.5 with formic acid) 0.5% high pH buffer B (0.1% Ammonium hydroxide, 99.9% acetonitrile). Over a 30 minute time period high pH buffer B was increased to 50%, followed by a 4 minute time period to increase high pH buffer B to 75%. The concentration of high pH buffer B (75%) was held for 4 minutes then reduced back down to 0.5%. Fifteen second fractions were collected for the duration of the gradient, the volume of each fraction was then decreased under vacuum.

### Mass Spectrometry

Peptides were identified by RP-LC-MS/MS on either a QStar® XL, QStar® Elite or Tof/Tof 5800 mass spectrometer (AB Sciex, UK). For the QStar® XL dried peptide fractions were re-suspended in 180 µl of 2% (v/v) acetonitrile/0.1% (v/v) formic acid. For each analysis one third of the peptide sample was loaded onto a 15 cm reversed phase C18 column (75 µm inner diameter) packed with C_18_ PepMap100 (3 µm, 100 Å) using an LC Packings (Amsterdam, Netherlands) UltiMate™ pump and separated as described previously (R.D.Unwin et al. Molecular Cellular Proteomics. 2005, 4, pp924–935). Briefly, peptides were separated over a 120 min solvent gradient from 5.9% (v/v) acetonitrile/0.1% (v/v) formic acid to 41% (v/v) acetonitrile/0.1% (v/v) formic acid on-line to a QStar® XL mass spectrometer (AB Sciex, UK). Data was acquired using an information dependant acquisition (IDA) protocol where, for each cycle, the two most abundant multiply charged peptides (2^+^ to 4^+^) above a 20 count threshold in the MS scan with m/z between 400 and 2000 were selected for MS/MS. Each peptide was dynamically excluded (±50 mmu) for 1 minute.

For the QStar® Elite dried peptide fractions were re-suspended in 15 µl of 3% (v/v) acetonitrile, 0.1% (v/v) formic acid and 20 mM citric acid. For each analysis, 5 µl of the peptide sample was loaded onto a nanoACQUITY UPLC Symmetry C18 Trap, 5 µm, 180 µm×20 mm and flow was set to 15 µl/min of 3% (v/v) acetonitrile, 0.1% (v/v) formic acid and 20 mM citric acid for 5 min. Analytical separation of the peptides was performed using nanoACQUITY UPLC BEH C18 Column, 1.7 µm, 75 µm×250 mm. Briefly, peptides were separated over a 91 min solvent gradient from 3% (v/v) acetonitrile,0.1% (v/v) formic acid 20 mM citric acid to 40% (v/v) acetonitrile,0.1% (v/v) formic acid, 20 mM citric acid on-line to a QStar® Elite mass spectrometer (AB Sciex, UK). Data was acquired using an information dependant acquisition (IDA) protocol where, for each cycle, the four most abundant multiply charged peptides (2^+^ to 4^+^) above a 10 count threshold in the MS scan with m/z between 400 and 2000. Each peptide was dynamically excluded (±50 mmu) for 90 seconds.

For the Tof/Tof 5800 system dried peptide fractions were re-suspended in 180 µl of 2% (v/v) acetonitrile/0.1% (v/v) formic acid and a third of the sample loaded onto a trap column using a U3000 liquid chromatography system (Dionex, Sunnyvale, CA) and the peptides fractionated by a capillary RP C18 HPLC column (Acclaim PepMap C18, 3 µM 100Å) at a flow rate of 0.8 µl/min with a gradient of between 2–40% (v/v) acetonitrile, 0.1% (v/v) TFA over 80 minutes. The flow through was spotted onto a MALDI plate (AB SCIEX, UK) in 15 second fractions using an online Probot (Dionex, Sunnyvale, CA) with α-cyano-4-hydroxycinnamic acid mixing with the eluant to a final concentration of 1.25 mg/ml. Mass spectrometry was carried out on an AB SCIEX TOF/TOF 5800 (AB SCIEX, Framingham, USA) using 1000 shots for MS. MS/MS was carried out on the top 27 precursors with a S/N of higher then 8 using 4000 laser shots, a 2 Kv acceleration voltage and air as the collision gas.

### Data Analysis

Data was processed by a ‘Thorough’ search against the ensembl (FIXME reference www.ensembl.org) mouse database (release 58) containing 115,658 protein entries using ProteinPilot 3 software (Paragon version 3.0.0.0, 113442) with default settings including the allowance of one missed or nonspecific cleavage (AB SCIEX, Framingham, USA), MMTS and 8 plex iTRAQ fixed modifications. For a list of all variable modifications see ProteinPilot 3.

Phosphopeptides identified with a confidence of >20 were included in the analysis. ProteinPilot quantification was used for the proteome experiments. For the phosphopeptide experiments, inferred phosphopeptide entities were selected from the ProteinPilot search results. The calculation used by ProteinPilot to derive a weighted average ratio for proteins was used to derive an average ratio where multiple spectra were found for a given distinct phosphoentity (that is, a distinct sequence and set of modified amino acids). As the number of identifications for an entity is usually very low the p-value was calculated where possible but not used to determine a change.

### Microarray Analysis

RNA was prepared using TRIzol (Invitrogen), DNase treated then cleaned using MinElute RNeasy Clean up Kit (Qiagen) per manufacturers instructions. Transcriptome analysis was undertaken using murine GeneChip® Mouse Exon 1.0 ST Arrays by the CR-UK Affymetrix microarray facility (Paterson Institute, Manchester, UK; http://bioinformatics.picr.man.ac.uk/vice/Protocols.vice). All data were analysed as previously reported [14].

### Interactive Web-based Viewer of Data

An interactive web-based viewer for the data is available at http://www.scalpl.org/scope3/. A screen snap of the display is shown in figure 7b. This tool gives a clustered view of all proteins or phosphopeptides that were discovered (each represented by a hexagon). The clustering was performed using the GATE software [53]. The brightness of each hexagon indicates the significance of change determined for the protein or phosphopeptide and selected oncogene (red for up-regulation and green for down-regulation). The oncogene to which the comparison is made can be changed from the menu above the hexagon display. By hovering the pointer over a hexagon the effects of the different oncogenes can be quickly compared using the bar chart on the right side of the display. Further information from gene ontology and the change ‘calls’ can be overlaid on the display by selecting them from the list below the bar chart. Clicking on a hexagon displays more detailed information for that protein in the panel below.

## Supporting Information

Figure S1 **TGFβ inhibitor studies.** Control and leukaemogenic PTKs transfected Ba/F3 cells were cultured in the presence of 0, 0.5, 1.0, 5.0, 10 and 50 µM of the TGFβ receptor inhibitor LY364947 (Calbiochem, UK). Cell viability was assessed at 24, 48 and 72 hours by trypan blue exclusion.
(PDF)

Table S1 **All proteins identified.** Table showing all 3763 nuclear proteins identified across the three replicate experiments of Ba/F3 cells transfected with 6 oncogenic PTKs. The first four columns display protein and gene accessions, gene symbol and name. The next nine columns give information from the search results for the identification of the protein in each experiment (blanks if not identified). “Coverage” indicates what percentage of the protein sequence was identified. “Unique peptides” indicates the number of unique peptides discovered whilst “Peptides used” indicates the number of unique peptides used to derive the quantification. The remaining columns give, for each oncogene in turn, quantification ratios (against MSCV control) and associated p-values and error factors for each replicate.
(XLSX)

Table S2 **Change significance interval.** Table showing the ratios employed to define a change. These are the values that 95% of ratios for the internal replicate lie between. This “significance interval” is individually determined for each experimental run and attempts to account for the technical and biological variation seen in each run.
(DOCX)

Table S3 **Proteins changing as a result of expression of each oncogene.** Proteins defined as changing are shown for each oncogene separately. To be called as changing a protein must have a ratio outside the range in which 95% of protein ratios for the internal replicate are found and a p-value of 0.05 or less in the majority of experiments that a ratio is recorded and not changing in any internal replicate. The first four columns display protein and gene accessions, gene symbol and name. The “change” column gives a significance for the likelihood of all the ratios representing a change and whether this is an increase or decrease in protein expression. The next three columns indicate in how many replicates the observation was made and whether it was an increase or decrease. “Coverage” indicates the percentage of the protein sequence identified. “Unique peptides” indicates the number of unique peptides discovered whilst “Peptides used” indicates the number of unique peptides used to derive the quantification. The remaining columns give, for each oncogene in turn, quantification ratios (against MSCV control) and associated p-values and error factors for each replicate.
(XLSX)

Table S4 **All phosphopeptides identified.** Table showing all phospho peptides identified across the three replicate experiments. The first four columns give protein and gene accessions, gene symbol and name from which each peptide originates. The next two columns list the modification assigned to each peptide and the peptide sequence. The next six columns display a value for the confidence with which the peptide was matched (Confidence) and the number of matches used for the quantification (Observations Used) for each of the three replicate runs. The remaining columns give, for each oncogene in turn, quantification ratios (against MSCV control) and associated p-values and error factors for each replicate.
(XLSX)

Table S5 **Phosphopeptide spectra.** Table showing all phospho peptides identified across the three replicate experiments and their observed mass and charge. The first column shows which experiment the peptide was seen. The next two columns display a unique number identifying the spectrum and the peptide match to the spectrum. The next three provide the accession number, peptide sequence and cleavage information. The next columns display a value for the confidence with which the peptide was matched (Confidence). The next four columns provide information on the charge, precursor mass and whether the peptide was used in the quantification calculation and an explanation as to why it was excluded if not used in the quantification.
(XLSX)

Table S6 **Phosphopeptides changing as a result of expression of each oncogene.** Phosphopeptides defined as changing are shown for each oncogene. To be called as changing a peptide must have a ratio outside the range in which 95% of peptide ratios for the internal replicate are found in at least one experiments and not changing in any internal replicate. The first four columns give phosphopeptide sequence, protein accession, gene symbol and name from which each peptide originates. The “change” column gives a significance for the likelihood of all the ratios representing a change and whether this is an increase or decrease in peptide expression. The next three columns indicate in how many replicates the observation was made and whether it was an increase or decrease. The remaining columns then display for each replicate a value for the confidence with which the peptide was matched (Confidence), the number of matches used for the quantification (Spectra Used), a value for the confidence in the likelihood of all the ratios representing a change (Change) and quantification ratios (against MSCV control) and associated p-values and error factors.
(XLSX)
