# Correction: Identification of Nuclear Protein Targets for Six Leukemogenic Tyrosine Kinases Governed by Post-Translational Regulation

**DOI:** 10.1371/annotation/df82a4c0-2909-43a0-8a7f-5bd2ce9ca0bd

**Published:** 2013-06-27

**Authors:** Andrew Pierce, Andrew Williamson, Ewa Jaworska, John R. Griffiths, Sam Taylor, Michael Walker, Mark Aspinall O’Dea, Elaine Spooncer, Richard D. Unwin, Toryn Poolman, David Ray, Anthony D. Whetton

The name of the seventh author was given incorrectly. The correct name is: Mark Aspinall-O'Dea. 

