# Supplementary figures and images for: Identification of Nuclear Protein Targets for Six Leukemogenic Tyrosine Kinases Governed by Post-Translational Regulation
Source: PLoS One. 2012 Jun 22;7(6):e38928. doi: 10.1371/journal.pone.0038928 (PMC3382166; doi:10.1371/journal.pone.0038928)

Supplementary Figure 1

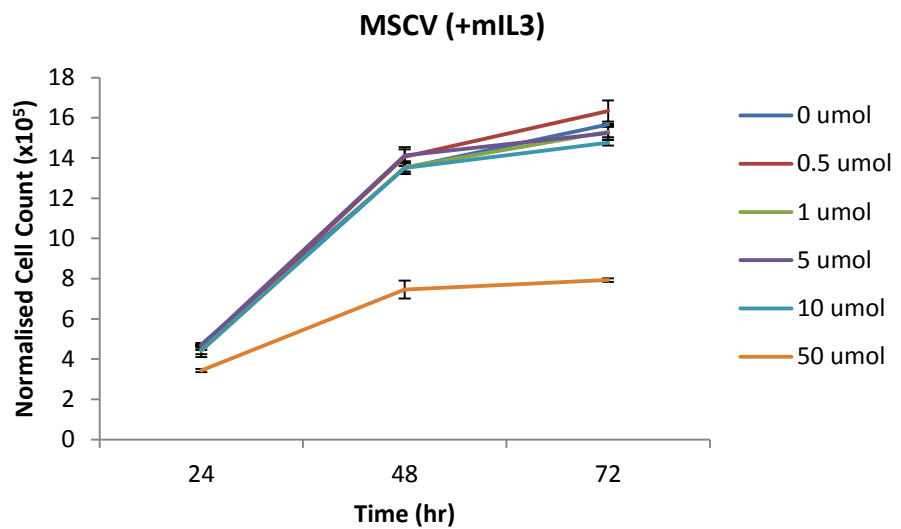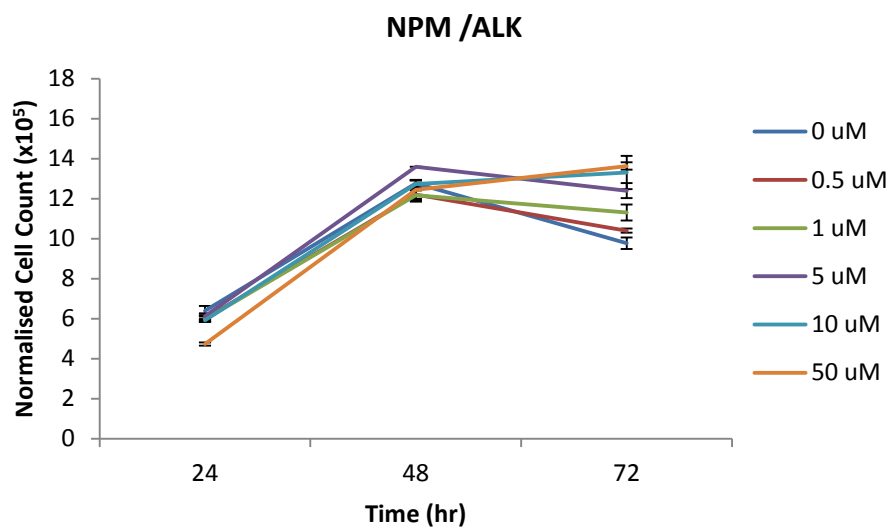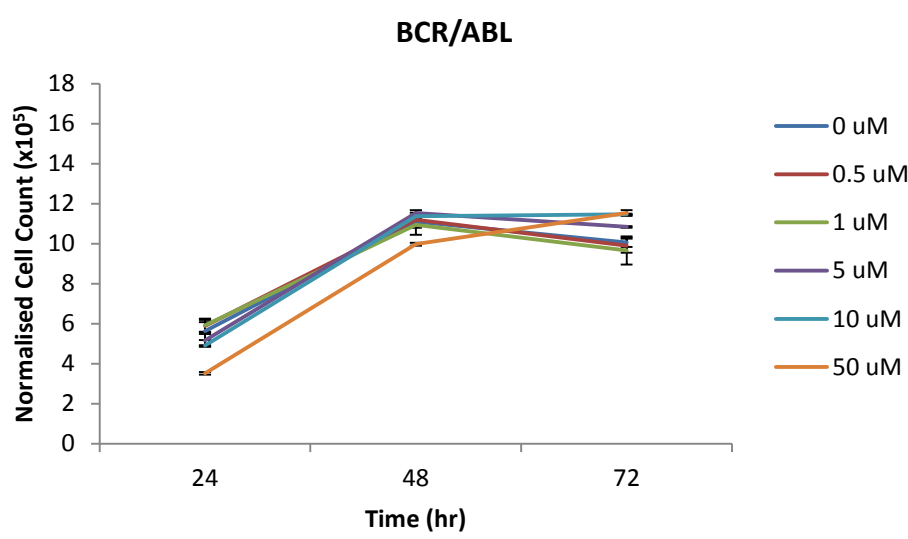

### TEL /PDGFRB

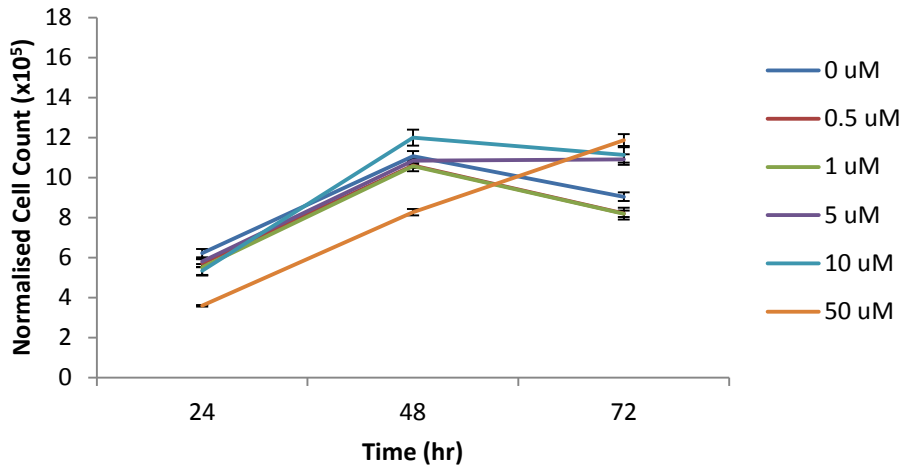

### FLT3 ITD

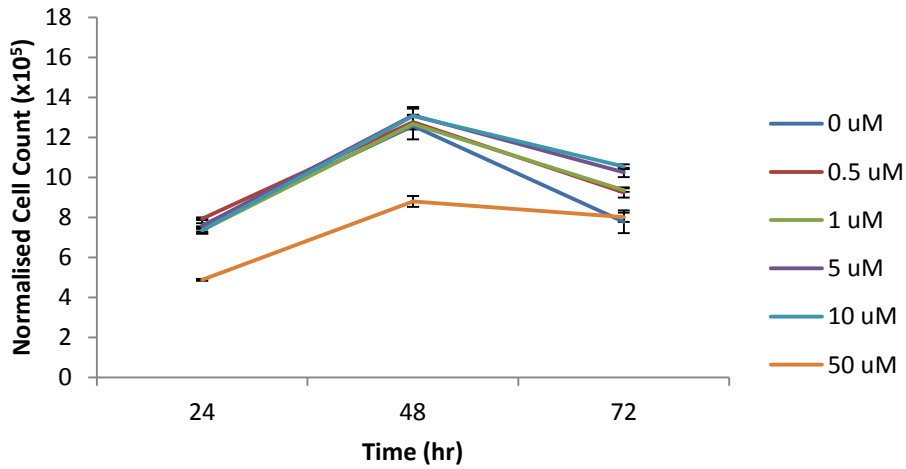

### KIT mutant

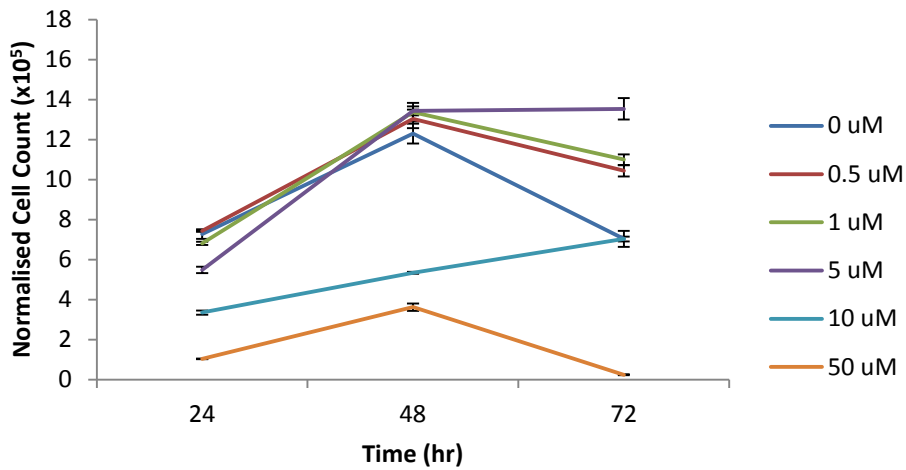

### FIP1/PDGF $\alpha$

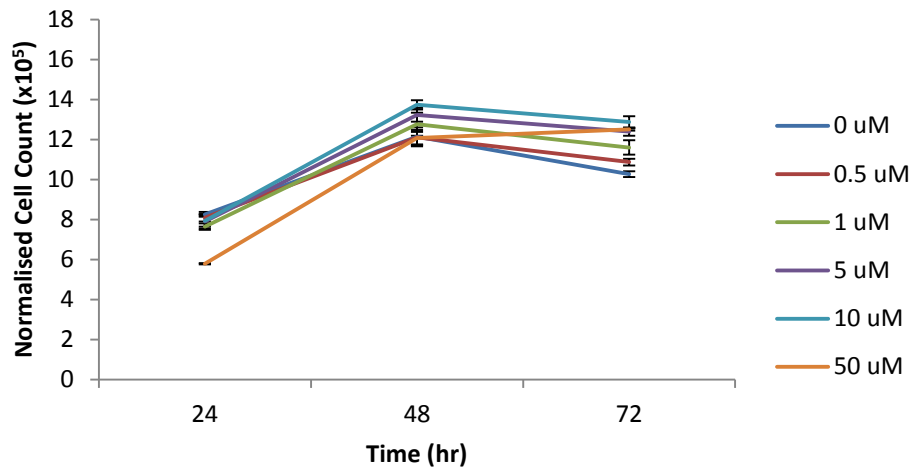

Supplement: Figure S1 — TGFβ inhibitor studies. Control and leukaemogenic PTKs transfected Ba/F3 cells were cultured in the presence of 0, 0.5, 1.0, 5.0, 10 and 50 µM of the TGFβ receptor inhibitor LY364947 (Calbiochem, UK). Cell viability was assessed at 24, 48 and 72 hours by trypan blue exclusion. (PDF) [file pone.0038928.s001.pdf]
