# Supplementary material for: Identification of Nuclear Protein Targets for Six Leukemogenic Tyrosine Kinases Governed by Post-Translational Regulation
Source: PLoS One. 2012 Jun 22;7(6):e38928. doi: 10.1371/journal.pone.0038928 (PMC3382166; doi:10.1371/journal.pone.0038928)
Supplement: Table S2 — Change significance interval. Table showing the ratios employed to define a change. These are the values that 95% of ratios for the internal replicate lie between. This “significance interval” is individually determined for each experimental run and attempts to account for the technical and biological variation seen in each run. (DOCX) [file pone.0038928.s003.docx]

|  | **95% significance interval defining a change** | | |
| --- | --- | --- | --- |
|  | **Run1** | **Run 2** | **Run 3** |
| **Proteome** | **>1.5 to < 0.7** | **>1.5 to < 0.7** | **>1.3 to < 0.7** |
| **Phosphoproteome** | **>2.3 to < 0.4** | **>1.7 to < 0.6** | **>1.4 to < 0.7** |
